# National dental policies and socio-demographic factors affecting changes in the incidence of periodontal treatments in Korean: A nationwide population-based retrospective cohort study from 2002–2013

**DOI:** 10.1186/s12903-016-0310-0

**Published:** 2016-11-05

**Authors:** Jae-Hong Lee, Jung-Seok Lee, Jung-Kyu Choi, Hye-In Kweon, Young-Taek Kim, Seong-Ho Choi

**Affiliations:** 1 Department of Periodontology, Daejeon Dental Hospital, Wonkwang University College of Dentistry, Daejeon, 35233 South Korea; 2 Department of Periodontology, Research Institute for Periodontal Regeneration, Yonsei University College of Dentistry, 50 Yonsei-ro, Seodaemun-gu, Seoul 03722 South Korea; 3 Department of Health Insurance Research, Ilsan Hospital, National Health Insurance Service, Goyang, 10444 South Korea; 4 Department of Periodontology, Ilsan Hospital, National Health Insurance Service, 100 Ilsan-ro Ilsan-donggu, Goyang, 10444 South Korea

**Keywords:** Cohort analysis, Dental insurance, Health insurance, Health services accessibility, Periodontal disease

## Abstract

**Background:**

Dental insurance coverage has recently expanded in Korea. The number of patients diagnosed with periodontal disease (PD), and the actual number of periodontally compromised patients has increased. However, few studies have investigated the relationships between the prevalence of periodontal disease and the incidence of PD treatment, dental insurance policies, and socio-demographic factors. To determine the incidence of periodontal treatments required, the comprehensive longitudinal data of the National Health Insurance Service were used. This study evaluated changes in the incidence of periodontal treatments, using data from the Korean National Health Insurance Cohort Database.

**Methods:**

A random stratified sample of 1,025,340 Korean patients was selected from National Health Insurance database, using 1,476 multistage samplings (of sex, age, and income level) for 12 years from 2002 to 2013. Chi-square analysis, and univariate, and multivariate logistic regression were used to evaluate the association of socio-demographic factors with the prevalence of PD and the incidence of periodontal treatment.

**Results:**

The incidence of periodontal treatment steadily and significantly increased, in both male and female participants, from 2002 to 2013. The increase was associated with socio-demographic factors and changes in national dental insurance policies. The incidence of periodontal treatment evaluated by age is influenced by the changes in national dental policies. These results suggest that the increase in patients diagnosed with PD reflects changes in dental policies and insurance benefits.

**Conclusions:**

This study confirms that national dental policies and socio-demographic factors are related to the incidence of periodontal treatments. The incidence of periodontal treatment is significantly related to the expansion of insurance coverage in South Korea.

## Background

The demands for medical care in modern societies are increasing. These demands result from increases in the aging population, household income, and education levels, and changes in health policies and the medical care systems [1]. Recently, the importance of oral-health-related quality of life has been recognized [2, 3]. Periodontal disease (PD) is a disease of the oral cavity, and a major cause of tooth loss in adults [4]. More than half of all adults are affected by some degree of PD, and 11 % of adults suffer from severe and advanced periodontitis [5]. In 2014, in South Korea, 12.9 million people (26 % of the current population) received treatment for PD. Periodontal disease is a highly prevalent disease, and is the second most common outpatient disease, after acute bronchitis [6].

Recent expansion of insurance coverage for dental treatment has resulted in an increase in the number of patients diagnosed with PD, and an increase in the identification of periodontally compromised patients [6]. The incidence of periodontal treatments is closely and consistently related to socio-demographic factors, including age, sex, race, household income, insurance, and health status, and living area [7, 8]. The severity of disease, and the incidence of periodontal treatments, is higher among those in disadvantaged groups, including, individuals who are older, poorer, less educated, and those living in rural areas [9, 10]. In addition, although there is no clear evidence, both public health policy and insurance availability are considered to have an impact on PD treatment efficacy and outcome [11–14].

Universal health care was implemented in Korean in 1989. By 2013, 97.1 % of the Korean population (51.34 million people) was covered by the mandatory social National Health Insurance Service (NHIS) [15]. Insurance for 49.99 million people is provided by NHIS, and the Medical Aid Program (MAP) insures an additional 1.46 million people. The dental health-care service was implemented after the medical-care service and it continues to expand. Major dental insurance policies presently cover the costs of sealant (first and second molars, from May 2013), full complete dentures (from July 2012), removable partial dentures (from July 2013), and dental implant treatment (from July 2014) for individuals aged 75 years and above. They also cover the cost of periodontal scaling (for individuals 20 years and above, from July 2013) [15]. Dental insurance coverage continues to expand and coverage for full and partial dentures, and dental implants is expected to be available by 2015 for individuals aged 70 years and above.

Several studies, such as the National Health and Nutrition Examination Survey (NHANES) from the United States, and the Adult Dental Health Survey from the United Kingdom, have investigated the epidemiology of PD [11, 16]. These reports are useful for assessing the prevalence of PD, and planning national dental services. However, these studies are based on cross-sectional surveys. This method not only limits the ability to assess changes in the incidence of periodontal treatments over time, but also cannot be used to identify the impact of dental insurance coverage on periodontal treatments. When compared with cross-sectional surveys, a longitudinal study can be used to assess changes in the incidence of periodontal treatments over time and to identify the impact of dental insurance coverage on periodontal treatments [17]. In South Korea, several cross-sectional studies, have investigated the association between PD, national dental policies, and socio-demographic factors. However, an observational cohort study has not been conducted [3, 18]. The Korean National Health Insurance Cohort Database (KNHICD) includes 12 years of accumulated individual socio-demographic factors, and is useful for evaluating changes in the incidence of periodontal treatments. Therefore, the aim of our study was to identify national dental policies, and socio-demographic factors, influencing the incidence of periodontal treatments. To accomplish this aim, we used the comprehensive data, accumulated from 2002 to 2013, in the KNHICD.

## Methods

### Study design and data source

Mandatory national health insurance in South Korea results in a database (DB) of more than one trillion individual cases, involving the entire Korean population of 50 million individuals, and includes data from the start-up of mandatory enforcement of insurance payment benefit in 1977. This DB includes information on qualifications, insurance, medical and dental records, checkups, rare incurable diseases, cancer, and long-term-care insurance services.

The NHIS made the KNHICD available for research purposes in 2013. This nationwide, population-based retrospective cohort study evaluated the association between treatments for PD, national dental policies, and socio-demographic factors of the South Korean population. A random stratified sample of one million Koreans was selected from the DB by the NHIS Big Data Steering Department, using 1,476-stage sampling (of sex, age, and household income level), for the 12 years from 2002 to 2013.

Effective from 2003, records that no longer existed, because of death or emigration, were replaced with records from newborns born in the same year. To protect privacy, the identification number of each patient was anonymized. All personal information is managed solely by NHIS and the request for raw data is offered only with given random identification codes.

### Ethics, consent, and permissions

The project was approved by the NHIS Ilsan Hospital, Goyang, Korea. An ethical approval was also obtained by the NHIS Ilsan Hospital (approval #2016-03-019).

### Study population clinical characteristics

The KCD-6 (Korean Classification of Disease; modified version of ICD-10 [International Classification of Disease]) guidelines were used to diagnose PD (acute periodontitis [K052]; chronic periodontitis [K053]; periodontosis [K054)]; other PD [K055], and unspecified PD [K056]) according to criteria proposed by the American Academy of Periodontology [19].

The study population was defined as patients who were diagnosed with PD, and treated with periodontal surgery, by a general dentist or a specialized dentist (e.g., periodontist), in 2003. Treatment was defined according to the following NHIS prescription codes: U1051/1052, periodontal flap operation (simple/complicated); U1071/1072, bone graft for alveolar bone defects (allogenic, xenogenic, or substitute bone graft/autogenous bone graft); or U1081–1083, guided tissue regeneration (without bone graft/allogenic, xenogenic, or substitute bone graft/autogenous bone graft).

### Definition of socio-demographic factors

Socio-demographic factors were identified from the KNHICD and categorized into the following five groups: sex, age, monthly income, health status, and living area. The groups were further divided (using random stratified analysis by the NHIS Big Data Steering Department) into smaller sub-groups. The factor sex had two groups (male and female participants). Age was divided into eight groups; those < 69 years were divided into 10-year interval groups and those > 70 years formed one group. Monthly household income was divided into 41 groups based on the monthly household income of the participants. It was classified into quintiles, with medical aid beneficiaries in the first-quintile group. Based on health status of the participants, three groups were formed as healthy, minor disability, and major disability (using the Handicapped Welfare Law). Finally, the factor living was divided into two groups on the basis of its population; < 50,000 residents was classified as rural and ≥ 50,000 residents classified as urban.

### Statistical analysis

Chi-square analysis, and univariate and multivariate logistic regression were used to evaluate the association between socio-demographic factors and the prevalence of PD. Odds ratios (ORs) and 95 % confidence intervals (CIs) were calculated in all assessments, and a *P* value of < 0.05 was considered indicative of statistical significance. Statistical analyses were performed using Statistical Analysis System (version 9.2, SAS Institute, Cary, NC, USA) by the Department of Health Insurance Research, Ilsan Hospital, NHIS.

## Results

### Characteristics of the study population

The baseline characteristics of the cohort are presented in Table 1. Of the 1,025,340 individuals included in the study, male (513,258) and female (512,082) patients were equally represented. Patients aged 20–49 years (526,891) accounted for 51.4 % of the population. Additionally, 97.0 % (994,627) of patients were in the NHIS (employees and self-employed); 97.3 % (998,030) were classified as having good health status; and 89.8 % (920,588) lived in urban areas.

**Table 1:** Baseline characteristics of the study cohort

	Males		Females		Total
	*n*	%	*n*	%	
Total	513258	50.1 %	512082	49.9 %	1025340
Age group (years)					
≤ 9	71921	14.0 %	64661	12.6 %	136582
10–19	74447	14.5 %	67495	13.2 %	141942
20–29	86355	16.8 %	84492	16.5 %	170847
30–39	95929	18.7 %	91788	17.9 %	187717
40–49	85646	16.7 %	82681	16.1 %	168327
50–59	48254	9.4 %	48862	9.5 %	97116
60–69	34353	6.7 %	41169	8.0 %	75522
≥ 70	16353	3.2 %	30934	6.0 %	47287
Household income^a^					
First quintile	70088	13.7 %	84513	16.5 %	154601
Second quintile	79071	15.4 %	78685	15.4 %	157756
Third quintile	103744	20.2 %	97450	19.0 %	201194
Fourth quintile	123727	24.1 %	118422	23.1 %	242149
Fifth quintile	136628	26.6 %	133012	26.0 %	269640
Insurance status					
MAP	13049	3.2 %	17664	3.4 %	30713
NHIS, employees	248048	48.3 %	248549	48.5 %	496597
NHIS, self-employed	252161	49.1 %	245869	48.0 %	498030
Health status^b^					
Healthy	494934	96.4 %	503096	98.2 %	998030
Major disability	5828	1.1 %	3633	0.7 %	9461
Minor disability	12496	2.4 %	5353	1.0 %	17849
Living area^c^					
Urban	460837	89.8 %	459751	89.8 %	920588
Rural	52421	10.2 %	52331	10.2 %	104752

*NHIS* National health insurance service, *MAP* Medical aid program

^a^Total of 41 groups classified into quintiles (with MAP beneficiaries in the first quintile)

^b^Classification based on the Handicapped Welfare Law in South Korea

^c^Classification based on rural (<50,000 residents) or urban (≥50,000 residents) living

### Incidence of periodontal treatments

**Fig. 1:**
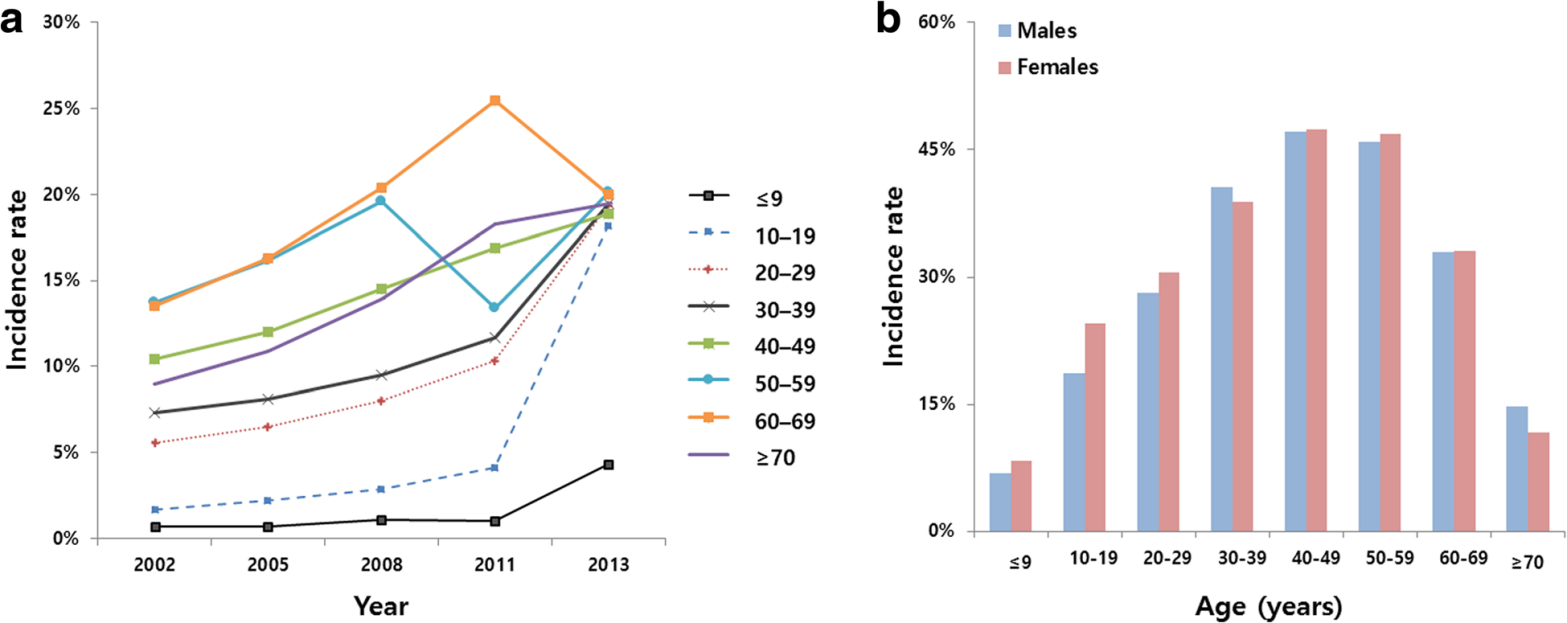
Age-specific 1-year incidence rates of periodontal treatment (**a**); and sex-specific accumulated incidence rates of periodontal treatments (**b**) during 2002–2013 in South Korea

Of the patients diagnosed with PD, 30.8 % (158,303) were male and 31.8 % (162,800) female. The incidence of periodontal treatments increased significantly from 2002 (*n* = 71,917; 7.0 %) to 2013 (*n* = 183,312; 18.1 %). In 2013, the incidence was higher in female patients (*n* = 91,856; 18.1 %), when compared to male patients (*n* = 91,456; 18.0 %). The incidence of periodontal treatments increased in all groups in 2013, except in patients 60–69 years of age. The 1-year incidence rate of PD among those aged 10–19 years increased from 2011 (*n* = 5,529; 4.1 %) to 2013 (*n* = 22,566; 18.2 %; Fig. 1a). From 2002 to 2013, the accumulated incidence of periodontal treatments peaked among patients 40–49 years of age, for both male (*n* = 40,426; 47.2 %) and female (*n* = 39.205; 47.4 %) patients. Among those 30–39 years of age and over 70 years of age, the accumulated incidence was higher in male patients than female patients. The accumulated incidence was higher in female patients for all other age groups (Fig. 1b).

**Fig. 2:**
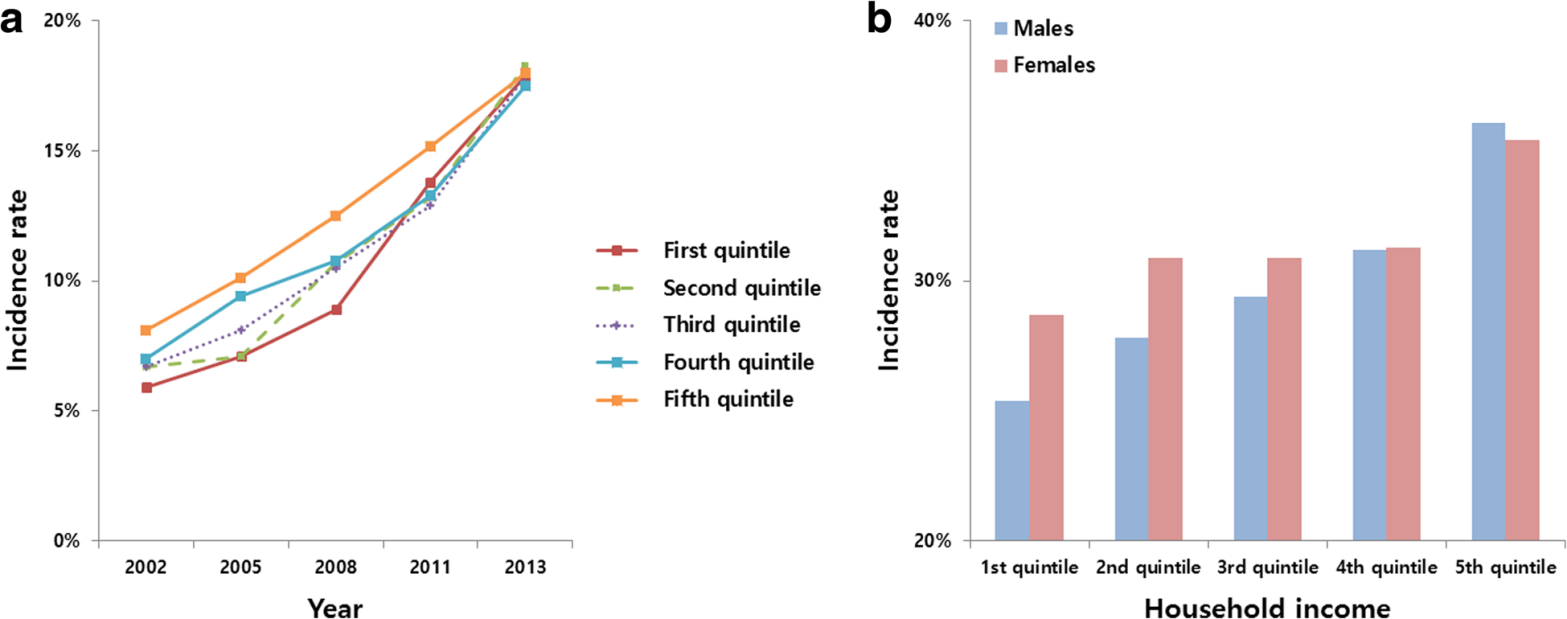
Household income-specific 1-year incidence rates of periodontal treatment (**a**); and sex-specific accumulated incidence rates of periodontal treatment (**b**) during 2002–2013 in South Korea

The incidence of periodontal treatments increased from 2002 to 2013; this increase was unrelated to household income level. The increase was greatest in the first quintile, from 5.9 % (*n* = 9,056) in 2002, to 17.9 % (*n* = 30,388) in 2013 (Fig. 2a). The accumulated incidence of periodontal treatments in the first quintile, which includes the MAP group (*n* = 42,102; 27.2 %), increased, and was similar to that in the fifth quintile (*n* = 96,393; 35.7 %). The accumulated incidence of periodontal treatments was higher in female patients (*n* = 24,277; 28.7 %), than male patients (*n* = 17,825; 25.4 %) in the first quintile, similar for both sexes in the fourth quintile (38,661; 31.2 % and 37,042; 31.3 %), and higher in male patients (*n* = 49,286; 36.1 %), when compared to female patients (*n* = 47,107; 35.4 %), in the fifth quintile (Fig. 2b).

**Fig. 3:**
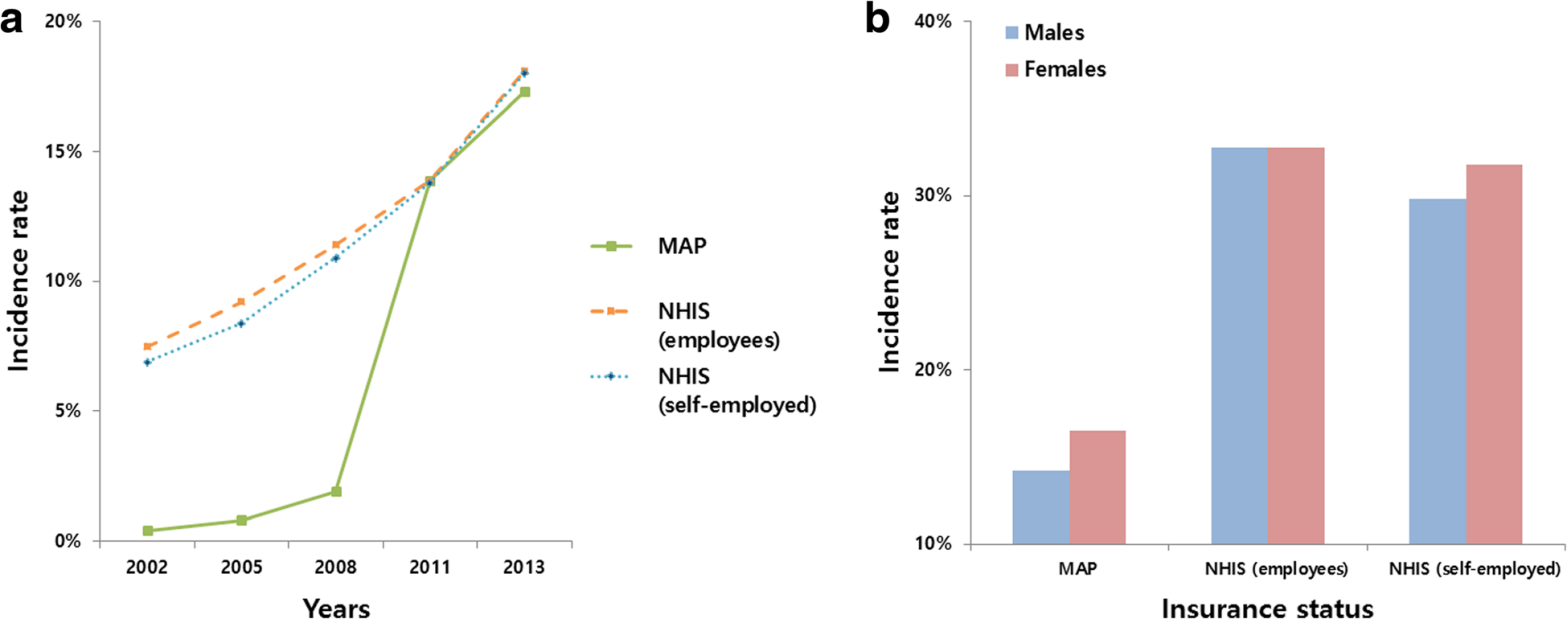
Insurance status-specific 1-year incidence rates of periodontal treatment (**a**); and sex-specific accumulated incidence rates of periodontal treatment (**b**) during 2002–2013 in South Korea. NHIS, National Health Insurance Service; MAP, Medical Aid Program

The incidence of periodontal treatments showed a tendency to increase in the NHIS group. In 2002, 7.5 % (37,368) of employees and 6.9 % (34,412) of self-employed individuals underwent periodontal treatments. In 2013, 18.1 % (124,315) of employees and 18.0 % (53,782) of self-employed individuals underwent treatment. However, from 2008 to 2011, the incidence of periodontal treatments increased sevenfold in the MAP group (*n* = 244; 1.9 % vs. *n* = 4,751; 13.9 %). In 2013 the period incidence in the MAP group was 17.3 % (*n* = 5,212), which was similar to that in the NHIS group (Fig. 3a). The accumulated incidence in the MAP group was 15.5 %, which was significantly lower than that in the NHIS group (32.8 % of employees and 30.8 % of self-employed). There was no difference between male and female patients in any of the groups (Fig. 3b).

**Fig. 4:**
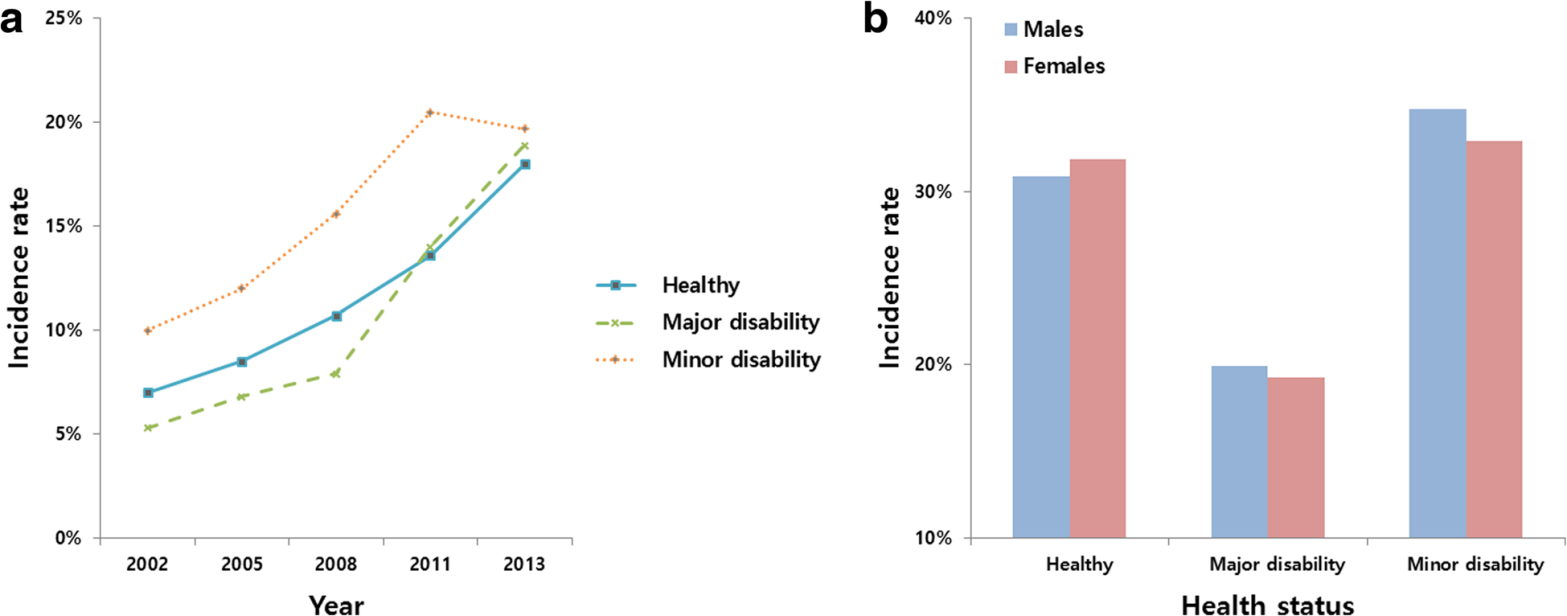
Health status-specific 1-year incidence rates of periodontal treatment (**a**); and sex-specific accumulated incidence rates of periodontal treatment (**b**) during 2002–2013 in South Korea

The overall incidence of periodontal treatments increased from 2002 to 2013, to 19.7 % (7,688) in the minor disability group (Fig. 4a). The accumulated incidence of periodontal treatments differed with health status, and was 19.7 % in the major disability group, 31.4 % in the healthy group, and 34.2 % in the minor disability group. There were no significant differences between men and women in any of these groups (Fig. 4b).

**Fig. 5:**
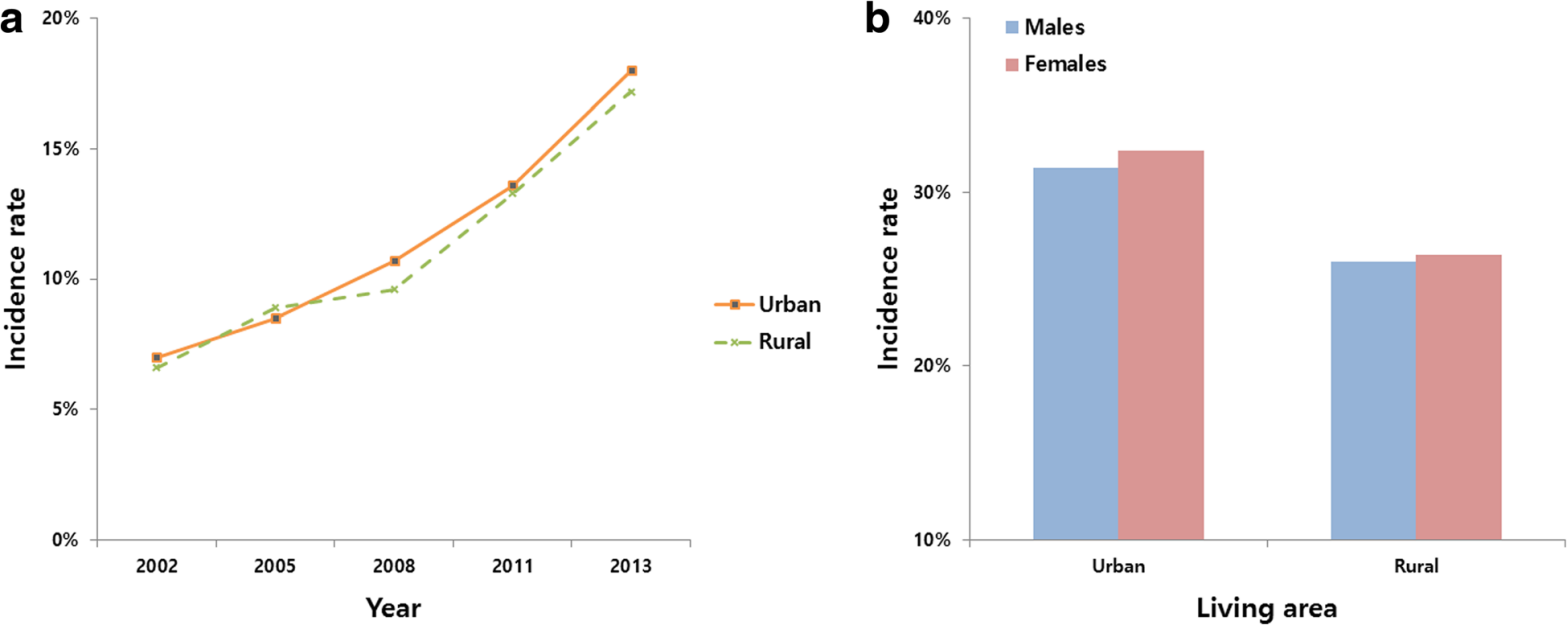
Living area-specific 1-year prevalence rates of incidence rates of periodontal treatment (**a**); and sex-specific accumulated incidence rates of periodontal treatment (**b**) during 2002–2013 in South Korea

The incidence of periodontal treatments increased from 2002 to 2013, for patients in both urban and rural areas. In 2013, the incidence was 18.0 % (170,124) for urban patients, and 17.2 % (13,188) for rural patients (Fig. 5a). The accumulated incidence of periodontal treatments was 31.9 % in urban areas and 19.7 % in rural areas. There was no significant difference between the sexes (Fig. 5b).

### Association of socio-demographic factors with periodontal treatments

The socio-demographic factors associated with periodontal treatments are shown in Table 2. Univariate analysis indicated that all socio-demographic factors investigated were significantly associated with periodontal treatments (*P* < 0.001); however, with multi-variant analysis, the only factor that was not statistically significant was sex (*P* = 0.513). Among all age groups, when using those ≤9 years of age as the reference, the OR for the incidence of periodontal treatments was highest among patients 40–49 years of age (OR = 9.58, 95 % CI = 9.36–9.81, *P* < 0.001). For household income, using the first quintile as the reference, the OR was 1.37 (95 % CI = 1.35–1.39, *P* < 0.001) for the fifth quintile. The incidence of periodontal treatments increased with household income. For insurance status, using the MAP group as the reference, the OR was 1.93 (95 % CI = 1.86–2.00, *P* < 0.001) for those in the NHIS (employees) group. For health status, using those in the healthy group as the reference, the major disability group had an OR of 0.61 (95 % CI = 0.58–0.64, *P* < 0.001) and the minor disability group had an OR of 0.94 (95 % CI = 0.91–0.97, *P* < 0.001). When using the urban-area group as the reference for living area, the rural-area group had an OR of 0.85 (95 % CI = 0.83–0.86, *P* < 0.001).

**Table 2:** Association of sociodemographic factors with periodontal treatments in univariate and multivariate analyses

	Univariate analysis				Multivariate analysis			
	OR		95 % CI	*P*	OR^a^		95 % CI	*P*
Sex								
Male	1.00	ref			1.00	ref		
Female	1.05		1.04–1.05	<0.001	1.00		1.00–1.01	0.513
Age group (years)								
≤ 9	1.00	ref			1.00	ref		
10–19	3.35		3.27–3.43	<0.001	3.50		3.41–3.58	<0.001
20–29	5.10		4.98–5.21	<0.001	5.19		5.07–5.32	<0.001
30–39	8.10		7.92–8.28	<0.001	7.72		7.55–7.90	<0.001
40–49	11.02		10.77–11.26	<0.001	9.58		9.36–9.81	<0.001
50–59	10.61		10.36–10.86	<0.001	8.39		8.18–8.60	<0.001
60–69	6.05		5.90–6.20	<0.001	4.67		4.54–4.80	<0.001
≥ 70	1.17		1.72–1.84	<0.001	1.64		1.58–1.70	<0.001
Household income								
First quintile	1.00	ref			1.00	ref		
Second quintile	1.11		1.09–1.13	<0.001	1.02		1.00–1.04	0.029
Third quintile	1.15		1.14–1.17	<0.001	1.10		1.08–1.12	<0.001
Fourth quintile	1.22		1.20–1.23	<0.001	1.18		1.16–1.20	<0.001
Fifth quintile	1.49		1.47–1.51	<0.001	1.37		1.35–1.39	<0.001
Insurance status								
MAP	1.00	ref			1.00	ref		
NHIS, employees	2.66		2.58–2.74	<0.001	1.93		1.86–2.00	<0.001
NHIS, self-employed	2.42		2.34–2.50	<0.001	1.65		1.59–1.71	<0.001
Health status								
Healthy	1.00	ref			1.00	ref		
Major disability	0.54		0.51–0.56	<0.001	0.61		0.58–0.64	<0.001
Minor disability	1.14		1.10–1.17	<0.001	0.94		0.91–0.97	<0.001
Living area								
Urban	1.00	ref			1.00	ref		
Rural	0.77		0.76–0.78	<0.001	0.85		0.83–0.86	<0.001

*OR* Odds ratio, *NHIS* National health insurance service, *MAP* Medical aid program

^a^OR, calculated by multivariate

## Discussion

Based on data in the KNHICD, our study shows that the incidence of periodontal treatments increased significantly from 2002 to 2013. The increase was seen in both male and female patients, and was due to both socio-demographic factors and the changes in national dental policies. Although there have been reports of sex differences in the incidence of periodontal treatments, this study found similar prevalence rates for both sexes, and that the prevalence for both sexes increased steadily. The incidence of periodontal treatments, according to age, have been influenced by changes in the national dental policies [16, 18]. From 2002 to 2011, the prevalence among individuals less than 20 years of age was similar to previously reported rates (5–8 %) [20, 21]. However, in 2013, the incidence of periodontal treatments among those 10 to 19 years of age increased by 18.2 %. This increase is likely attributable to expansion of insurance coverage for sealant treatment for those younger than 18 years of age, and to the improved accessibility to dental services, which resulted in an increase in the number of adolescents being diagnosed with periodontal disease.

The difference between the high-incidence age group and the low-incidence age group, excluding those less than 9 years of age, can be explained by limited accessibility to dental services [7, 22]. In 2013, with the expansion of dental insurance coverage to include dentures and implants the incidence of periodontal treatments was expected to increase for individuals over 70 years of age. The incidence of periodontal treatments was lower in individuals with severe disabilities, and those living in rural areas. Although previous studies report the incidence of periodontitis was higher in the elderly and low-income individuals, the present study did not confirm these results. Decreased accessibility to dental services, due to individual socio-demographic characteristics, and lifestyle risk factors, may explain the different results [22]. Factors such as SES, health behaviors, and general health problems are associated with tooth loss. The main socio-demographic factors associated with tooth loss are, older age, low income, and low education level. Other oral-health-related factors, such as smoking, inadequate brushing habits, noncompliance with dental treatment, and poor dental knowledge, are also associated with tooth loss. General health factors related to tooth loss include coronary heart disease, diabetes, high blood sugar level, and limited physical activity [7, 8].

South Korea is an ethnically and culturally a homogeneous nation. In 2014, the population included only 3 % foreigners, mostly Mongoloid Chinese [23]. Therefore, unlike multiethnic countries, there are few racial barriers present in South Korea; hence, economic barriers are considered to be the most important factors restricting access to dental health-care services [24]. To overcome the SES disparities, various public health and insurance policies have been implemented. However, our study found that the disadvantaged groups still experience difficulties in accessing dental treatment. The incidence of periodontal treatments was 25.4 % among male patients in the first quintile of household income, and 36.1 % among those in the fifth quartile; the corresponding rates for female patients were 28.7 % and 35.4 %. This difference is attributed to an excessive number of voluntary non-reimbursable dental treatments, even when patients are covered by health insurance.

Prior to 2008, the incidence of periodontal treatments was significantly lower in the MAP group when compared to the NHIS group (comprising employees and self-employed). The incidence of periodontal treatments has increased in the MAP group since 2008, and it was similar (14.1 %) to that in the NHIS group. This increase is likely associated with 2008 changes in public health and insurance policies, which included the secondary poor population into the NHIS group. Policy changes were aimed at moderating the rapid increases in public expenditure on the MAP; however, these changes had no effect on the incidence of periodontal treatment in the MAP group.

Periodontal disease is characterized by chronic, irreversible, and progressive periodontal tissue destruction. Early detection of PD tends to be difficult, due to the absence of symptoms and lack of pain in early disease [25]. In addition, although still controversial, several studies have found a relationship between PD and comorbidities, such as cardiovascular disease, diabetes, premature birth, erectile dysfunction, and Alzheimer’s disease [26, 27]. The incidence of periodontal treatments in the present study increased from 2002 to 2013. This increase is likely due to improvements in accessibility to dental health-care service, rather than an increase in the number of periodontally compromised patients.

The paradigm for dental insurance policies has changed in recent years, to focus on prevention and early diagnosis. Specifically, since 2013, there has been an expansion of dental insurance coverage to include periodontal scaling, dentures, and dental implants and it is expected that the incidence of periodontal treatments will continue to increase. Our study did not evaluate dental records or include detailed diagnostic codes, which limited the ability to diagnose the severity of PD. In addition, although there are still many uninsured voluntary non-reimbursable treatments for dental care, these data are not included in the KNHICD. These issues represent a limitation of the analysis performed in the present study. Nevertheless, in order to evaluate changes in the incidence of periodontal treatments, we used a precisely extracted cohort from the KNHICD, which includes data from all citizens of South Korea, as well as information from medical institutions. The results of this study suggest that national dental policies and socio-demographic factors are closely related to changes in the incidence of periodontal treatments, suggesting that this study will be useful in establishing dental policies that decrease the incidence of PD, and enhance the oral health of the entire population of South Korea.

## Conclusions

Based on data in the KNHICD from 2002 to 2013, this study shows that the incidence of periodontal treatments has increased steadily, in both male and female patients, in South Korea. This study shows that the national dental policies and socio-demographic factors are clearly related to the incidence of periodontal treatments. Specifically, expansion of insurance coverage for dental treatments appears to be related to the increased incidence of periodontal treatment.

## Abbreviations

DB: Database
ICD: International classification of disease
KCD: Korean classification of disease
KNHICD: Korean national health insurance cohort database
MAP: Medical aid program
NHANES: National health and nutrition examination survey
NHIS: National health insurance service
OECD: Organization for economic cooperation and development
PD: Periodontal disease
SES: Socioeconomic status
